# Neuroimaging of Basal Ganglia in Neurometabolic Diseases in Children

**DOI:** 10.3390/brainsci10110849

**Published:** 2020-11-12

**Authors:** Justyna Paprocka, Magdalena Machnikowska-Sokołowska, Katarzyna Gruszczyńska, Ewa Emich-Widera

**Affiliations:** 1 Department of Pediatric Neurology, Faculty of Medical Science in Katowice, Medical University of Silesia, 40-055 Katowice, Poland; marekwidera@wp.pl; 2 Department of Diagnostic Imaging, Radiology and Nuclear Medicine, Faculty of Medical Science in Katowice, Medical University of Silesia, 40-055 Katowice, Poland; magdams@onet.pl (M.M.-S.); kgruszczynska@poczta.onet.pl (K.G.)

**Keywords:** basal ganglia abnormalities, children, neurometabolic disease

## Abstract

Diseases primarily affecting the basal ganglia in children result in characteristic disturbances of movement and muscle tone. Both experimental and clinical evidence indicates that the basal ganglia also play a role in higher mental states. The basal ganglia can be affected by neurometabolic, degenerative diseases or other conditions from which they must be differentiated. Neuroradiological findings in basal ganglia diseases are also known. However, they may be similar in different diseases. Their assessment in children may require repeated MRI examinations depending on the stage of brain development (mainly the level of myelination). A large spectrum of pathological changes in the basal ganglia in many diseases is caused by their vulnerability to metabolic abnormalities and chemical or ischemic trauma. The diagnosis is usually established by correlation of clinical and radiological findings. Neuroimaging of basal ganglia in neurometabolic diseases is helpful in early diagnosis and monitoring of changes for optimal therapy. This review focuses on neuroimaging of basal ganglia and its role in the differential diagnosis of inborn errors of metabolism.

## 1. Introduction

**Figure 1:**
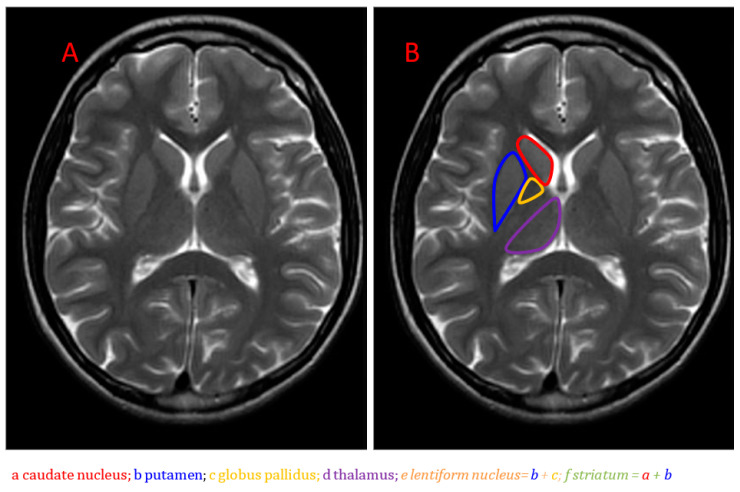
Brain anatomy on MRI, the basal ganglia on the T2-weighted image, axial plane (**A**,**B**).

Anatomically, the basal ganglia are deep symmetrical structures of the gray matter. Most authors consider the following structures to be part of the basal ganglia: caudate and lentiform nuclei, thalamus, subthalamic nuclei and substantia nigra [1]. The lentiform nucleus consists of the inner globus pallidus and outer putamen (putamen belongs to the structure of the striatum together with a distinct caudate nucleus) (Figure 1).

The basal ganglia are involved in both motor and non-motor functions, including higher order cognition, social interactions, speech and repetitive behaviors [2,3,4,5,6]. The first and main clinical symptoms of diseases with basal ganglia involvement are disorders of movement and muscle tone [1].

Due to subtle differences between the white and gray matter, magnetic resonance is the imaging method of choice because density changes on computed tomography (CT) scans are mostly unnoticed. Imaging of the basal ganglia in children is challenging due to infantile and childhood brain development and significant physiological changes in the appearance and signal of brain tissues.

**Figure 2:**
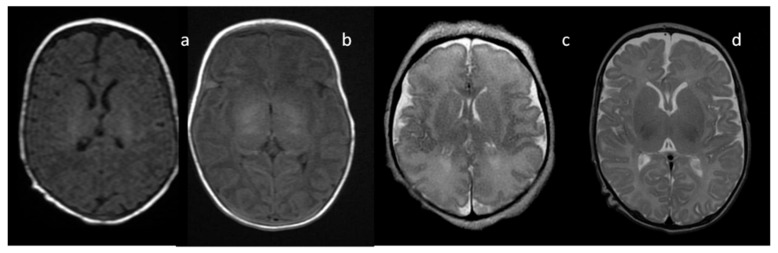
Infant brain MRI—no differences in signal intensity between the basal ganglia and the surrounding structures before the completion of myelination: (**a**,**b**) axial T1-weighted images (T1-WI) in a neonate with a hyperintense signal in the posterior internal capsule; (**c**) axial T2-weighted images (T2-WI) at the 36th week of gestation; (**d**) axial T2-WI in a neonate.

**Figure 3:**
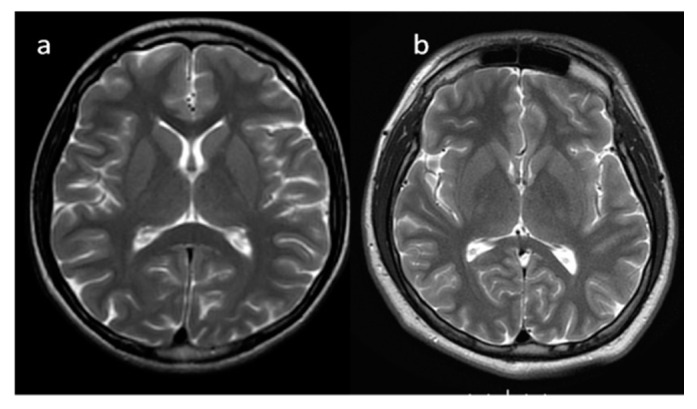
(**a**,**b**). Completion of myelination on brain MRI and differences in signal intensity between the basal ganglia and the surrounding brain tissues (axial T2-WI).

**Figure 4:**
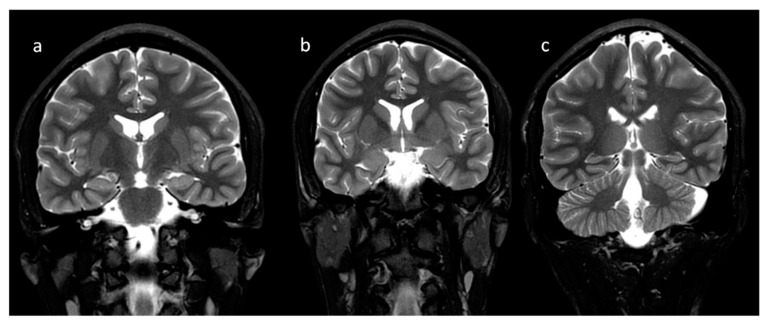
Completion of myelination on brain MRI and differences in signal intensity between the basal ganglia and the surrounding brain—coronal T2-WI; putamen and caudate nucleus head (**a**,**b**); thalamus and caudate body (**c**).

In neonates, the white matter is iso- to hypointense compared to the gray matter on T1-weighted images (T1-WI) and iso- to hyperintense on T2-weighted images (T2-WI). Therefore, it is difficult to differentiate between the white and gray matter in the basal ganglia due to the lack of myelination in the internal capsule (Figure 2). Signal intensity at the posterior limb is changed, which results in a consecutive increase in T1 signal intensity (at 0–1 month) and a decrease in T2 signal intensity (at 0–3 months) [2] (Figure 3). Myelination of the anterior limb is a longer process (increase in signal intensity on T1-WI between the 2nd and 3rd month and decrease on T2-WI between the 7th and 11th month) [2] (Figure 4).

Location of changes in the deep nuclei depends on different degrees of sensitivity to different insults.

Many diseases, including some neurometabolic disorders, change signal intensity from the basal ganglia. Their assessment may require repeated examinations depending on the stage of brain development (mainly the level of myelination). Basal ganglia imaging consists of multiple sequences including T1-WI, T2-WI, T2 * GRE/SWI, diffusion-weighted imaging (DWI) and contrast-enhanced imaging. These options are commonly used in clinical practice.

Changes in the basal ganglia signal might be of hyper-, hypo- or mixed intensity on T1-WI, hyper-, hypo- or mixed intensity on T2-WI or MRI-related artefacts (hemoglobin, calcium, iron, DWI) with the presence or the lack of gadolinium contrast enhancement. Of note, the imaging studies provide the description of the distribution of pathology and symmetry or asymmetry of lesions [7]. Advanced imaging methods such as positron emission tomography (PET), diffusion tensor imaging (DTI), perfusion-weighted imaging (PWI) and MR spectroscopy (MRS) provide a deeper insight into basal ganglia with an additional analysis of the metabolites. Therefore, PET examination is more useful in the assessment of adults, mainly due to its high radiation dose. Pediatric patients can benefit from safer and more advanced MRI techniques. Diffusion tensor tractography (DTI) provides more precise information on motor disorders. Some diseases might cause both hyper- and hypointensity, e.g., “the eye of the tiger” sign or the Parkinson variant [7,8,9].

In clinical practice, MRI of the head is used as the initial examination of all patients suspected of having neurometabolic diseases with the basic protocol containing the sequences T1-WI, T2-WI, T2 * GRE/SWI, diffusion-weighted imaging (DWI). In some patients, MRS examination, due to a detailed analysis of the presence of metabolites typical for a given disease, allows for its confirmation or exclusion. DTI analysis is rarely used—more often in research work than in everyday practice. In the diagnosis of neurometabolic diseases in children, the use of PET as a method that exposes a high dose of radiation is avoided.

Precise identification of calcium, hemosiderin, iron or metals is not always possible and might be easier with special sequences (e.g., T2 * GRE).

A large spectrum of pathological changes in the basal ganglia in many diseases is caused by their vulnerability to metabolic abnormalities [7,8]. Physiological high activity with high glucose and oxygen uptake, rich vascular supply, a large amount of mitochondria and chemical substances make the basal ganglia very sensitive to metabolic, chemical or ischemic trauma [8].

Most metabolic diseases and some vascular disorders (e.g., central venous infarction) cause symmetrical basal ganglia changes [7,9]. Asymmetry of the pathology usually leads to the diagnosis of infarction or ischemia, an infectious or neoplastic process, which are not discussed in this paper as they are beyond the scope of the article [7,8]. Location of abnormal signal intensity in the basal ganglia (Table 1) aids in the differential diagnosis [7,8,9]. Contrast administration is useful in infarction, encephalitis and neoplastic conditions. Most changes in metabolic diseases are not enhanced following gadolinium administration. The use of advanced techniques (PWI, DTI, MRS) renders MRI a powerful tool. 

## 2. MRI Abnormalities of the Basal Ganglia Depending on the Anatomical Localization

### 2.1. Globus Pallidus

Abnormal signal of globus pallidus involvement may be isolated or coupled with other abnormalities. For example, in pantothenate kinase-associated neuropathy, the globus pallidus T2 hypointensity or T2 hypointensity with central T2 hyperintensity may coexist with cerebral or cerebellar atrophy [7,8,9,10]. 

Isolated globus pallidus MRI signal change with T2 prolongation may be seen in the following disorders:

- succinic semialdehyde dehydrogenase (SSADH);
- methylmalonic acidemia;
- isovaleric acidemia;
- pyruvate dehydrogenase deficiency (due to mutation in the dihydrolipoamide acetyltransferase gene and in the E2 component);
- guanidinoacetate methyltransferase (GAMT) deficiency;
- carbon monoxide poisoning;
- chronic phase of kernicterus [7,8,9,10].

Globus pallidus hyperintensity on T2-WI or FLAIR with subcortical white matter demyelination and involvement of the cerebellar dentate nuclei may suggest Kearns–Sayre syndrome and L-2-hydroxyglutaric aciduria. In urea cycle disorders, hyperintensity of the globus pallidus on T2-WI with T2 prolongation of the insula and the perirolandic cortex can be detected [10].

Hyperintensity of the globus pallidus on T1-WI with:

- normal T2 signal intensity indicates chronic hepatic disease;
- hyperintensity on T2-WI indicates acute hyperbilirubinemia, systemic lupus erythematosus and hemolytic-uremic syndrome, which is most likely if there is associated edema involving the external and extreme capsules and the claustrum [7,8,9,10].

Globus pallidus and diffuse white matter involvement including the subcortical, deep and periventricular regions raises the suspicion of Canavan diseases [7,8]. 

In maple syrup urine disease (MSUD), a hyperintense signal with diffusion restriction is noted in the globus pallidus and the thalamus [9]. Corresponding changes in the internal capsules, cerebral peduncles, semioval center, dorsal pons and cerebellar white matter are also seen. MRI spectroscopy demonstrates a peak at 0.9 ppm characteristic of MSUD representing branched-chain ketoacids, which may facilitate the diagnosis [7,10]. However, in the early stages of MSUD, methylmalonic acidemia, cyanide or carbon monoxide toxicity or the diffuse white matter changes are not seen on MRI [10]. 

Contrary to semioval center white matter T2 hyperintensity found in the diseases described above, globus pallidus and subcortical white matter involvement with sparing of the periventricular white matter suggests a later phase of Kearns–Sayre syndrome or L-2-hydroxyglutaric aciduria [9].

### 2.2. Caudate and Putamen (Striatum)

Caudate and putamen abnormal signal is found in:

- mitochondrial disorders (Leigh syndrome, mitochondrial encephalopathy with lactic acidosis and stroke-like episodes (MELAS);
- glutaric aciduria;
- propionic acidemia;
- Wilson disease;
- juvenile Huntington disease;
- molybdenum cofactor deficiency;
- isolated sulfite oxidase deficiency;
- hypoxic-ischemic injury;
- hypoglycemia;
- hypomyelination with atrophy of the basal ganglia and cerebellum [7,8,9,10].

Additionally, other changes in the brain tissue are also found:

- glutaric aciduria type 1 with striatal hyperintensity is associated with enlarged subarachnoid spaces, particularly in the anterior Sylvian fissures, and hyperintense signal changes on T2-WI (suspicion of delayed maturation) in the thalamus, substantia nigra, nucleus dentatus, the central tegmental tract and the supratentorial white matter;
- isolated sulfite oxidase deficiency causes rapidly progressive multicystic encephalomalacia of the cerebral white matter;
- striatum calcification in Cockayne disease;
- in GM1, abnormalities of the posterior putamen are seen on brain MRI [7,8,10].

### 2.3. Thalamus

Primary involvement of the thalamus with hyperintensity on T1-WI and hypointensity on T2-WI on MRI may be found in GM1 and GM2 gangliosidosis [7,8,9,10]. In Krabbe disease, apart from thalamic involvement, abnormal T2 hyperintensity within the corticospinal tract is common [7,9].

Other disorders with thalamic hyperintense signal on T2-WI include:

- mitochondrial disorders typically with putaminal changes;
- Wilson disease with putaminal involvement;
- Canavan disease with globus pallidus abnormalities;
- nonmetabolic diseases often involving the thalamus (profound neonatal asphyxia with the damage to the ventrolateral thalamus, posterior putamen and the perirolandic cortex);
- acute necrotizing encephalopathy (ANE) with hyperintensity in the thalamus and dorsal brainstem on T2-WI [10].

## 3. Basal Ganglia MRI Involvement Depending on the Disease

Table 2 shows neuroimaging abnormalities in thalamus, pallidum and putamen in different neurometabolic diseases. The putamen is the most common localization of lesions.

## 4. Neurometabolic Disorders 

### 4.1. Mitochondrial Disorders

The prevalence of metabolic stroke in myoclonic epilepsy with ragged red fibers (MERRF) is even 60%, whereas in mitochondrial myopathy, encephalopathy, lactic acidosis and stroke-like episodes (MELAS), it is 84–99%. Even in 13% of children, MELAS and MERRF have been associated with calcification of the basal ganglia [11,12].

In the literature, the neuroimaging spectrum ranges from classical Leigh syndrome with symmetrical lesions in the basal ganglia and/or brainstem to structural abnormalities including cerebellar hypoplasia and corpus callosum dysgenesis [11,12,13]. Due to advances in genomic technology, mainly in next-generation sequencing (NGS) and whole-exome sequencing (WES), the genetic spectrum of Leigh syndrome has broadened and, so far, 75 genes have been identified both in mitochondrial (30%) and nuclear DNA [11,12,13]. Leigh syndrome is one of the most common mitochondrial disorders. 

In the rare mitochondrial encephalomyelopathy (Alpers disease), a “pulvinar sign”—bilateral symmetric pulvinar (mediodorsal thalamic nucleus) T2 high signal relative to the signal intensity of other deep gray matter nuclei and cortical—has been described [11,12,13]. This symptom is non-specific; it may occur in other diseases such as Creutzfeldt–Jacobs disease (CJD), postinfectious encephalitis and cat-scratch disease [12,13]. Differentiation is based on clinical data, including age, and other underlying lesions in the brain.

Stroke-like episodes or metabolic strokes are related to neurological deficits due to biochemical abnormalities in focal brain regions that are not connected with ischemic or hemorrhagic causes. Apart from mitochondrial defects, stroke-like episodes also occur in aminoacidopathies, urea cycle defects, lysosomal storage disorders or organic acidemias [14,15]. Almuqbil et al. reported on a patient with propionic aciduria with basal ganglia changes on brain MRI several years after the resolution of neurological symptoms [15]. MRI, angio-MRI and MRI spectroscopy are useful tools in differentiating between vascular and nonvascular factors. 

### 4.2. Neurodegeneration with Brain Iron Accumulation (NBIA)

This group of diseases is characterized by abnormal iron accumulation in the basal ganglia, leading to different progressive movement and developmental disorders. The subtypes of Neurodegeneration with Brain Iron Accumulation (NBIA) are connected to specific gene disorders. There are four main types including pantothenate kinase-associated neurodegeneration (PKAN), PLA 2G6-associated neurodegeneration (PLAN), mitochondrial-membrane protein associated neurodegeneration (MPAN) and beta-propeller protein associated neurodegeneration (BPAN). There are also other rare, familial forms of NBIA [16].

The radiological pattern in NBIA is described as the “eye of the tiger sign”. Iron storage is evident in the anteromedial part of the globus pallidus as central hyperintensity on T2-WI with the surrounding area of hypointensity [8].

Iron deposits may be also observed in Parkinson disease, atypical parkinsonian disorders, multiple sclerosis or Friedreich ataxia [10]. The presence of iron deposits in the basal ganglia is age-dependent and is not found at birth, which is crucial for the differential diagnosis [10]. MRI changes may precede clinical symptoms. 

### 4.3. Biotin-Thiamine-Responsive Basal Ganglia Disease (BTBGD) 

Biotin-thiamine-responsive basal ganglia disease (BTBGD), also known as thiamine transporter-2 deficiency, is an autosomal recessive disorder caused by *SLC19A3* gene mutation [17]. In BTBGD, episodes of recurrent encephalopathy (confusion, seizures, dystonia, ataxia, dysphagia, supranuclear facial palsy, external ophthalmoplegia) are triggered by febrile illness or stress [17,18]. Neuroradiologically, it is characterized by bilateral necrosis and severe edema within the basal ganglia in acute stages. The most typical MRI abnormalities include central destruction of the caudate heads and partial or complete loss of the putamen with diffusion restriction and sparing of the globus pallidus [16,17]. Alfhadel et al. reported diffuse cortical, subcortical and white matter involvement in acute phase next to basal ganglia pathologies on 18 patients [19,20]. Similarly, Kaseem et al. in their study on 15 patients observed coexisting changes in the mesencephalon, cerebral cortical and subcortical regions and thalamus in 80% of cases [21]. In 53%, patchy deep white matter affection was found, and the cerebellum was involved in 13.3% [21]. After thiamine and biotin therapy, resolution of changes was found in the mesencephalon, white matter and cortex. Kamasak et al. confirmed a typical involvement of the putamen and caudate with diffusion restriction [22]. In BTBGD, treatment with high doses of biotin and thiamine may influence the disease progression and seizure activity [22].

### 4.4. Pyruvate Dehydrogenase Deficiency (PDHD)

Pyruvate dehydrogenase deficiency (PDHD) results in early-onset encephalopathy with X-linked recessive inheritance. Newborns usually present with severe lactic acidosis and subsequently develop significant neurological defects similar to those observed in patients with Leigh syndrome [10,11,12]. Children with later-onset forms may present with paroxysmal neurological episodes, often precipitated by fever or exercise and frequently associated with psychomotor retardation and pyramidal tract signs [23]. Brain MRI mostly shows bilateral cystic lesions of the putamen and the brain parenchyma, coexisting with corpus callosum abnormalities, asymmetric hydrocephalus and small pons [23]. In less severe forms, basal ganglia lesions seen on MRI might be reversible with treatment, as shown by follow-up MRI.

### 4.5. Glutaric Aciduria (GA) Type 1

Typical cases are characterized by early progressive macrocephaly and/or hypotonia. Although the imaging pattern is not characteristic, widening of the Sylvian fissures with associated macrocephaly should suggest searching for GM1 [24,25,26]. Additional changes in the striatum (putamen and caudate) were typically found in the acute stage. The globus pallidus is affected less frequently than striatum. However, changes in cerebellar dentate nuclei may be present, as in the case of L-2-hydroxyglutaric aciduria [24,25,26]. 

### 4.6. Gangliosidosis (GM1 and GM2)

All gangliosidoses affect the volume of the cerebrum and cerebellum. Movement disorders, including tremor, generalized or focal dystonia, chorea and parkinsonism, are present in 30–50% of late-onset (juvenile and adult) forms, and more volumetric changes of brain structures in these forms were reported, with thalamic hyperintensities (infantile form) [7,8,9,10].

Infantile (type 1) and juvenile (type 2) forms cause signal abnormalities of the basal ganglia [10,27]:

- in GM1, hyperintensities of posterior putamen and globi pallidi on T1-WI with susceptibility effects on T2-WI were found;
- in GM2, thalamic hyperintensity on T1-WI, mixed striatum signal intensity on T2-WI, hypointense white matter on T2-WI (Tay–Sachs syndrome: hypointense on T2-WI (ventral thalamus) hyperintense on T2-WI (dorsal thalamus); Sandhoff disease: T2 hypointense thalami).

Changes in deep nuclei are more typical of infantile GM2, whereas juvenile and adult forms present with cerebellar hypo/atrophy.

### 4.7. Wilson Disease 

Wilson disease, the effect of ceruloplasmin deficiency, results in copper storage in the liver and subsequentially in the thalamus, putamen and brain stem. The abnormal signal at the level of brain stem is referred to as the “giant panda face” (on T2-WI: symmetrical hyperintensity in tegmentum, and comparatively low-preserved signal in red nuclei, pars reticularis and superior colliculi) and/or “miniature panda face” (on T2-WI: hyperintense aqueduct entrance with relatively hypointense central tegmental tracts) [28,29]. 

### 4.8. In Methylmalonic Aciduria (MMA) 

An increasing signal intensity on MRI is observed, typically with selective necrosis. A better insight into the basal ganglia pathology is provided by MRS. The NAA/Cr and NAA/Cho ratios in methylmalonic aciduria are lower than in healthy children [14,30]. 

### 4.9. Fabry Disease 

Fabry disease can manifest radiologically as a vast range of unspecific changes—hence the need for close clinical diagnostic workup. T1 hyperintensity of the lateral pulvinaris is thought to be pathognomonic [31].

### 4.10. Canavan Disease (CD) 

Deep nuclei (globi pallidi and thalami) are typically affected by Canavan disease (CD), whereas the striatum and dentate nuclei are spared. MRI also reveals bilateral symmetric white-matter hyperintensity on T2-WI, including involvement of the subcortical arcuate fibers [32]. It can be observed in the white matter of the cerebellum and brain stem and, particularly, in the subcortical white matter [32]. The disease is the only known metabolic disorder which causes an increase in N-acetylaspartate levels on MR spectroscopy.

## 5. Differential Diagnosis 

Unfortunately, deep nuclei are affected in many other diseases, which requires extended differential diagnosis. 

At the early stage of brain development, it is difficult to differentiate between hypoxic-ischemic encephalopathy and metabolic diseases.

Due to preterm birth, the thalamus is suggested to be the most vulnerable deep gray matter structure [33,34,35,36]. Limited neuroimaging data suggest that the damage to the basal ganglia and the thalamus may occur within 10 min following the hypoxic-ischemic insult [35]. 

Phacomatosis is typically found on MRI, but it may cause diagnostic problems at the early stage. Increased focal areas of signal intensity (FASI) in the deep nuclei occur progressively with age, which results in the need for regular follow-up [37].

The blood supply to the basal ganglia comes from the lenticulostriate arteries arising from anterior and middle cerebral arteries. The globus pallidus and the putamen are structures with large numbers of mitochondria, which may predispose them to generalized injuries. 

Radiological changes in the basal ganglia are similar to those found in vascular diseases. Of note, infarcts can also occur in children with a completely different background.

Neuroinfections are another group to be considered in the differential diagnosis. In the case of congenital TORCH infection, toxoplasmosis and cytomegalovirus (CMV) have a predilection for calcifications found also in the basal ganglia on CT and microcephaly, which results in brain volume loss [37,38,39].

Herpes simplex virus does not have a predilection for the basal ganglia. However, it can trigger autoimmune limbic encephalitis or vasculitis (mostly of lenticulostriate arteries) sometimes involving the basal ganglia. From the *Herpesviridae* family, particularly Epstein–Barr virus infection frequently involves the basal ganglia [40].

Autoimmune brain disorders may also present with basal ganglia pathology. Acute disseminated encephalomyelitis (ADEM), or an immune-mediated demyelinating CNS disorder, is clinically defined by acute multifocal neurological deficits including encephalopathy [41]. Apart from reversible white matter and spinal cord lesions, ADEM involves the thalami and basal ganglia [42]. Acute necrotizing encephalopathy type 1 and biotin-thiamine responsive basal ganglia disease may give a similar neuroradiological picture of basal ganglia lesions [43]. Basal ganglia disorders are also reported in some neuropsychiatric conditions (e.g., Sydenham chorea) caused by the immune response to past streptococcal infection [44]. The basal ganglia lesions are also related to many diseases, including encephalitis syndromes (e.g., encephalitis lethargica) and systemic autoimmune diseases (e.g., systemic lupus) [42,43].

Cerebral palsy (CP) is associated with a profound hypoxic insult. Approximately 70% of patients with extrapyramidal dystonic cerebral palsy present with changes in the basal ganglia or/and the thalamus [45,46,47]. For instance, the globus pallidus is the most vulnerable part of the basal ganglia in neonates with kernicterus. Approximately 15–20% of patients with dystonic CP present with the damage to the lentiform nuclei, followed by putamen injury [45,46,47].

After carbon monoxide toxic exposure (CO intoxication), damage to the globus pallidus occurs immediately, whereas the cerebral white matter damage occurs within hours [48]. The hypothesis about selective vulnerability of the globus pallidus may be connected with the mechanism (ischemia or hypoxemia), duration, severity and suddenness of damage [48,49]. Additionally, the location of the globus pallidus between two sources of blood supply is also important [49]. 

Hypoparathyroid patients can present with basal ganglia calcifications (BGC) on MRI, which may pose problems related to the differential diagnosis (the presence of focal or generalized seizures, the tonic-clonic type) [50]. 

Abnormalities in the basal ganglia were found in some psychiatric disorders (e.g., attention deficit hyperactivity disorder; ADHD). The prefrontal cortex and the basal ganglia (particularly the caudate nucleus) exhibit increased volumes [51,52,53,54]. 

Computed tomography is not a sensitive method for white-gray matter differentiation. However, it is the method of choice for calcium detection and the assessment of basal ganglia calcifications (BGC), which is the most common site of calcifications in the central nervous system. However, they can occur also in the gray and white matter junction, cerebellar parenchyma, the thalamus and dentate nucleus [55,56]. In general population, the prevalence of BGC is not well established and is estimated at 2–12.5% [55,56]. BGC may occur as a primary/idiopathic disease or a secondary symptom of the condition leading to brain calcifications. The pattern of basal ganglia calcifications may occur in [55,56]:

- chromosomal abnormalities: SLC20A2 8p11.21 PDGFRB 5q32 PDGFB 22q13.1 XPR1 1q25.3 MYORG 9p13.3;
- primary or secondary forms of the following: hypoparathyroidism, secondary hypoparathyroidism, infections (brucellosis, AIDS, toxoplasmosis, TORCH complex), autoimmune diseases (SLE);
- other conditions such as pseudohypoparathyroidism, Cockayne syndrome I and II, Aicardi–Goutières syndrome, mitochondrial diseases (MELAS, MERRF), Coats’ syndrome, toxic exposure to carbon monoxide, lead or other metals, neuroferritinopathy and NBIA.

Even 33% of HIV-infected children present with calcifications of the bilateral putamen and globus pallidus before the age of 10 months [55,56].

Physiological and age-related calcifications are usually bilateral and faint and involve the globus pallidus and other regions such as the pineal gland, falx and choroid plexus [55,56]. They may be an incidental finding in 15–20% of asymptomatic patients undergoing CT [55,56]. According to some researchers, susceptibility-weighted images (SWI), which are a fully velocity-compensated 3D gradient-echo (GE) MRI sequence, are able to definitely confirm calcification [57].

Radiological findings (location and pattern of calcifications) may be useful in differentiation between physiological and pathological findings. In Aicardi–Goutieres syndrome, calcifications are more frequently small and punctuate (mostly seen in the putamen, pallidus and thalamus) and occur in the deep and periventricular white matter [55,56,57].

Another very interesting but rare example of secondary brain calcifications is mutation in RAS-related protein 39B potentially linked with juvenile parkinsonism with early bilateral calcification of the globus pallidus [58]. 

Additional problems related to the differential diagnosis in the modern world are related to toxic changes (particularly drug-related changes) [59,60,61].

Vigabatrin treatment commonly used in infantile spasms may cause transient hyperintensity on T2-WI and restricted diffusion in the thalamus, globus pallidus, dentate nucleus and cerebral peduncles [59]. Additionally, in children with total parenteral nutrition, hypermanganesemia may provoke a bilateral and symmetrical increase in signal intensity in the globi pallidi, subthalamic nuclei and anterior pituitary gland on T1-WI only [60,61].

The use of illicit drugs (e.g., cocaine, heroin) or immunosuppressant therapy may also be complicated by acute and chronic basal ganglia changes, including infarcts [61]. 

This review focuses on basal ganglia changes which may be a part of a wide spectrum of different pediatric diseases: basal ganglia diseases (BGD) of different origin, white matter diseases (WMD) with basal ganglia involvement or inborn errors of metabolism with basal ganglia involvement. 

**Figure 5:**
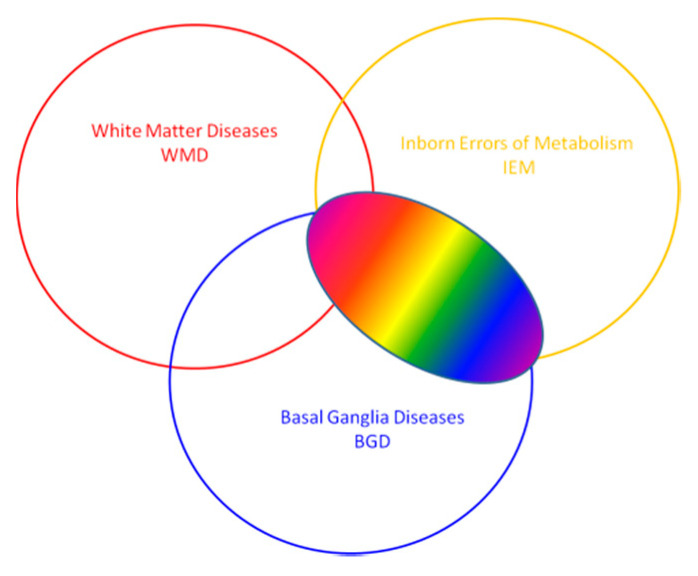
Focus of the review—neuroimaging of basal ganglia in neurometabolic diseases in children—involvement of basal ganglia in inborn diseases of metabolism, often with white matter involvement (three spectra of pathologies).

During the radiological analysis of diseases involving the basal ganglia, possible white matter abnormalities should also be assessed (Figure 5). Not all neurometabolic diseases affect the basal ganglia. On the other hand, the differential diagnosis of radiological changes in deep structures includes a wide group of diseases with a different background—not neurometabolic. Hence, an attempt to create a flow chart dealing only with changes in the basal ganglia would have to cover the whole group.

**Figure 6:**
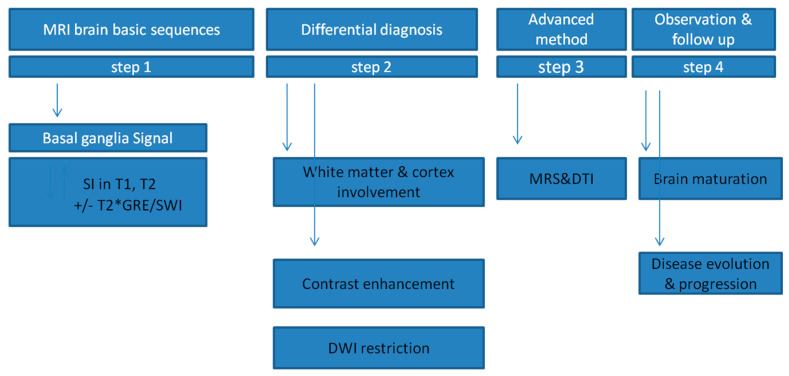
Scheme of radiological assessment of basal ganglia neuroimaging changes in neurometabolic diseases. Legend: SI—signal, GRE—gradient-echo/sequence, MRS—magnetic resonance spectroscopy, DTI—diffusion tensor imaging, DWI—diffusion-weighted imaging.

A diagram describing the radiological assessment of changes in the basal ganglia in neurometabolic diseases was proposed (Figure 6).

The authors have created a diagram of the steps of the radiological assessment of changes in the basal ganglia in neurometabolic diseases:

- Step 1

In the standard brain MRI protocol, signal disturbances are assessed in the form of increased or decreased signal intensities in the basic T1 and T2 sequences, which allows for initial differential diagnosis according to Table 1. The changes are also correlated with their anatomical location (Table 2). T2 * GRE/SWI sequences are used to assess possible calcifications, blood metabolites and the presence of certain metals (Mn, Cu, Fe); therefore, they are necessary in the diagnosis of changes in the deep nuclei.

- Step 2

The evaluation of white matter and cortex involvement is used to exclude or confirm coexisting changes. Moreover, in this group of diseases, some lesions spread to the fibers of the motor pathways. During this stage of the analysis, as part of the differential diagnosis, contrast enhancement is assessed each time—sporadic, atypical in neurometabolic diseases of the basal ganglia. DWI restriction in the gray matter of the subcortical nuclei occurs in some neurometabolic diseases only in their acute phase, which allows for the next step in differential diagnosis.

- Step 3

Diffusion tensor tractography (DTI) is particularly useful for the correlation of clinically identified motor disorders with changes in motor pathways. Magnetic resonance spectroscopy (MRS) is an advanced method for assessing the presence of metabolites in areas of signal disturbance, which may refine the diagnostic direction. The topic for further analysis would be the use and usefulness of MRS in the clinical suspicion of a neurometabolic disease without disturbance in the signal in the basal sequences.

- Step 4

Observation and follow-up in neurometabolic diseases is often a necessary part of a long diagnostic process in children due to two factors. The first factor is the brain maturation process. Abnormal signals may only be noticeable against the maturing/myelinating white matter. The second factor is the development of the disease. Radiological changes may initially be unnoticeable or subtle, appearing over time, and the unusual pattern of basal ganglia neuroimaging abnormalities, only in follow-up examinations, may prompt the correct diagnosis. Hence, the assessment of disease evolution and progression is in many cases crucial for diagnosis.

## 6. Summary

Basal ganglia abnormalities are found in a large spectrum of diseases with different etiologies. The biochemical and genetic diagnostics of neurometabolic disorders may be time-consuming. Early diagnosis in some diseases may prompt implementation of life-saving treatment such as biotine in BTBGD [21]. 

MRI is the gold standard for assessment of basal ganglia changes, whereas CT is useful for detection of calcification. Some basal ganglia abnormalities on MRI can be highly suggestive of a specific disease or a metabolic defect while others need further examination. 

Additional difficulty in infants is due to the development and continuous myelination during the first two years of life, which results in delayed diagnosis. First MRI examination is often normal for age. However, repeated follow-up imaging can provide more information. Even abnormal early MRI does not necessarily result in precise diagnosis, and follow-up MRI is needed to assess the direction of changes.

Pathologies in metabolic diseases are not stable and usually progress over time. Some diseases may be controlled with therapy, while MRI can monitor disease progression or stability.

Assessment of anatomical changes on MRI has been fundamental for diagnosis, differential diagnosis and follow-up. Newer techniques such as MRS, DTI and perfusion imaging offer some hope for a deeper insight into function and pathophysiological abnormalities in diseases involving the basal ganglia.

Knowing age-dependent signal changes, the pattern recognition of MRI findings and further correlation between radiological, clinical and genetic changes may allow faster initial diagnosis, thus leading to earlier and more precise treatment options.

## Figures and Tables

**Table 1:** Abnormal MR signal characteristics in particular sequences (T2-WI, T1-WI, DWI); metabolic diseases are given in bold [7,8,9].

Basal Ganglia MRI Signal Intensity	Disease
T2 hyper = increased signal on T2-WI	Hypoxic-ischemic encephalopathy (early) CO toxicity Kernicterus Mitochondrial diseases (e.g., Leigh disease) Infantile bilateral striatal necrosis Hypoglycemia Infarction Rasmussen encephalitis NF1 Myelinolysis Intoxications
T2 hypo = decreased signal on T2-WI	(acute) Deoxyhemoglobin Hypoxia
T1 hyper = increased signal on T1-WI	Gadolinium concentration after chelate administration Hypoxic-ischemic encephalopathy (late) CO toxicity NF1 Manganese toxicity (subacute) Methemoglobin Liver failure
T1 hypo = decreased signal on T1-WI	Infarction Infantile bilateral striatal necrosis
DWI restriction = increased signal on DWI with corresponding decreased signal on apparent diffusion coefficient (ADC) maps	Infarction Hypoxic-ischemic encephalopathy CO toxicity Mitochondrial diseases Encephalitis of different origin Wilson disease

T2-WI: T2-weighted images, T1-WI: T1-weighted images, DWI: diffusion-weighted imaging.

**Table 2:** Basal ganglia neuroimaging abnormalities in neurometabolic diseases [10].

Disease	Thalamus	Pallidum	Putamen
Respiratory chain disorders	**+**	**+**	**+**
Biotin-thiamine-responsive basal ganglia disease			**+**
Pyruvate dehydrogenase deficiency	**+**	**+**	**+**
Coenzyme Q10 deficiency			**+**
Mitochondrial thiamine pyrophosphate transporter			**+**
Cerebrotendinous xanthomatosis		**+**	
Alpha-methylacyl-CoA racemase deficiency	**+**		
GM1 gangliosidosis			**+**
Fabry disease	**+**		
Methylmalonic aciduria		**+**	
Succinic semialdehyde dehydrogenase deficiency		**+**	
Urea cycle disorders		**+**	
Glutaric aciduria type 1			**+**
Wilson disease	**+**	**+**	**+**
Aceruloplasminemia	**+**	**+**	**+**
Neuroferritinopathy		**+**	**+**
Pantothenate kinase-associated neurodegeneration		**+**	
Infantile neuroaxonal dystrophy		**+**	
Hypermanganemia		**+**	

## References

[1] Anderson J.C., Costantino M.M., Stratford T. (2004). Basal ganglia: Anatomy, pathology, and imaging characteristics. Curr. Probl. Diagn. Radiol..

[2] Seger C.A. (2008). How do the basal ganglia contribute to categorization? Their role in generalization, response selection, and learning via feedback. Neurosci. Biobehav. Rev..

[3] Shohamy D., Myers C.E., Kalanithi J., Gluck M.A. (2008). Basal ganglia and Dopamine Contributions to Probabilistic Category Learning. Neurosci. Biobehav. Rev..

[4] Riva D., Taddei M., Bulgheroni S. (2018). The neuropsychology of basal ganglia. Eur. J. Paediatr. Neurol..

[5] Mink J.W. (2018). Basal ganglia mechanisms in action selection, plasticity and dystonia. Eur. J. Pediatr. Neurol..

[6] Beudel M., Macerollo A., Brown M., Chen R. (2020). The role of the basal ganglia in somatosensory-motor interactions: Evidence from neurophysiology and behaviour. Front. Hum. Neurosci..

[7] Zuccoli G., Yannes M.P., Nardone R., Bailey A., Goldstein A. (2015). Bilateral symmetrical basal ganglia and thalamic lesions in children: An update. Neuroradiology.

[8] Hegde A.N., Mohan S., Lath N., Tchoyoson L. (2011). Differential Diagnosis for Bilateral Abnormalities of the Basal Ganglia and Thalamus. RadioGraphics.

[9] Pols T., de Vries L.S., Salamon A.S., Nikkels P.G.J., Lichtenbelt K.D., Mulder-de Tollenaer S.M., van Wezel-Meijler G. (2019). Symmetrical Thalamic Lesions in the Newborn: A Case Series. Neuropediatrics.

[10] Saudubray J.-M., Cazorla A.G., Saudubray J.-M., Baumgartner M.R., Walter J. (2016). Specific Neurosensorial, Neurophysiological and Neuroradiological Signs and Symptoms (at any Age). Inborn Metabolic Diseases, Diagnosis and Treatment.

[11] Baertling F., Klee D., Haack T.B., Prokisch H., Meitinger T., Mayatepek E., Schaper J., Distelmaier F. (2016). The many faces of paediatric mitochondrial disease on neuroimaging. Childs Nerv. Syst..

[12] Jde Beaurepaire I., Grévent D., Rio M., Desguerre I., de Lonlay P., Levy R., Dangouloff-Ros V., Bonnefont J.P., Barcia G., Funalot B. (2018). High predictive value of brain MRI imaging in primary mitochondrial respiratory chain deficiency. Med. Genet..

[13] Martikainen M.H., Ng Y.S., Gorman G.S., Alston C.L., Blakely E.L., Schaefer A.M., Chinnery P.F., Burn D.J., Taylor R.W., McFarland R. (2016). Clinical, Genetic, and Radiological Features of Extrapyramidal Movement Disorders in Mitochondrial Disease. JAMA Neurol..

[14] Fraser J.L., Venditti C.P. (2016). Methylmalonic and propionic acidemias: Clinical management update. Curr. Opin. Pediatr..

[15] Almuqbil M., Chinsky J.M., Srivastava S. (2019). Metabolic strokes in propionic acidemia: Transient hemiplegic events without encephalopathy. Child Neurol. Open.

[16] Hogarth P. (2015). Neurodegeneration with brain iron accumulation: Diagnosis and management. J. Mov. Disord..

[17] Schneider S.A. (2016). Neurodegeneration with brain iron accumulation. Curr. Neurol Neurosci. Rep..

[18] Salomão R.P., Pedroso J.L., Gama M.T., Dutra L.A., Maciel R.H., Godeiro-Junior C., Chien H.F., Teive H.A., Cardoso F., Barsottini O.G. (2016). A diagnostic approach for neurodegeneration with brain iron accumulation: Clinical features, genetics and brain imaging. Arq. Neuropsiquiatr..

[19] Alfadhel M., Almuntashri M., Jadah R.H., Bashiri F.A., Al Rifai M.T., Al Shalaan H., Al Balwi M., Al Rumayan A., Eyaid W., Al-Twaijri W. (2013). Biotin-responsive basal ganglia disease should be renamed biotin-thiamine-responsive basal ganglia disease: A retrospective review of the clinical, radiological and molecular findings of 18 new cases. Orphanet. J. Rare. Dis..

[20] Kassem H., Wafaie A., Alsuhibani S., Farid T. (2014). Biotin-responsive basal ganglia disease: Neuroimaging features before and after treatment. AJNR.

[21] Kamaşak T., Havalı C., İnce H., Eyüboğlu İ., Çebi A.H., Sahin S., Cansu A., Aydin K. (2018). Are diagnostic magnetic resonance patterns life-saving in children with biotin-thiamine-responsive basal ganglia disease?. Eur. J. Paediatr. Neurol..

[22] Algahtani H., Ghamdi S., Shirah B., Alharbi B., Algahtani R., Bazaid A. (2017). Biotin-thiamine-responsive basal ganglia disease: Catastrophic consequences of delay in diagnosis and treatment. Neurol. Res..

[23] Patel K.P., O’Brien T.W., Subramony S.H., Shuster J., Stacpoole P.W. (2012). The spectrum of pyruvate dehydrogenase complex deficiency: Clinical, biochemical and genetic features in 371 patients. Mol. Genet. Metab..

[24] Harting I., Neumaier-Probst E., Seitz A., Maier E.M., Assmann B., Baric I., Troncoso M., Mühlhausen C., Zschocke J., Boy N.P. (2009). Dynamic changes of striatal and extrastriatal abnormalities in glutaric aciduria type I. Brain.

[25] Boy N., Mühlhausen C., Maier E.M., Heringer J., Assmann B., Burgard P., Dixon M., Fleissner S., Greenberg C.R., Harting I. (2017). Proposed recommendations for diagnosing and managing individuals with glutaric aciduria type I: Second revision. Additional individual contributors. J. Inherit. Metab. Dis..

[26] Mohammad S.A., Abdelkhalek H.S., Ahmed K.A., Zaki O.K. (2015). Glutaric aciduria type 1: Neuroimaging features with clinical correlation. Pediatr. Radiol..

[27] Renaud D., Brodsky M. (2016). GM2-Gangliosidosis, AB Variant: Clinical, Ophthalmological, MRI, and Molecular Findings. JIMD Rep..

[28] Yu X.E., Gao S., Yang R.M., Han Y.Z. (2019). MR Imaging of the Brain in Neurologic Wilson Disease. AJNR.

[29] Członkowska A., Litwin T., Chabik G. (2017). Wilson disease: Neurologic features. Handb. Clin. Neurol..

[30] De Benedictis F.M., de Benedictis D. (2014). The value of neuroimaging in the assessment and follow-up of early-onset methylmalonic aciduria and homocystinuria. Mol. Genet. Metab. Rep..

[31] Cocozza S., Russo C., Pontillo G., Pisani A., Brunetti A. (2018). Neuroimaging in Fabry disease: Current knowledge and future directions. Insights Imaging..

[32] Israni A.V., Mandal A. (2017). Canavan disease with typical brain MRI and MRS findings. Neurol. India..

[33] Mastrangelo M., Di Marzo G., Chiarotti F., Andreoli C., Colajacomo M.C., Ruggieri A., Papoff P. (2019). Early Post-cooling Brain Magnetic Resonance for the Prediction of Neurodevelopmental Outcome in Newborns with Hypoxic-Ischemic Encephalopathy. J. Pediatr. Neurosci..

[34] Imai K., de Vries L.S., Alderliesten T., Wagenaar N., van der Aa N.E., Lequin M.H., Benders M.J.N.L., van Haastert I.C., Groenendaal F. (2018). MRI Changes in the Thalamus and Basal Ganglia of Full-Term Neonates with Perinatal Asphyxia. Neonatology.

[35] Rennie J., Rosenbloom L. (2011). How long have we got to get the baby out? A review of the effects of acute and profound intrapartum hypoxia and ischaemia. Obstet. Gynaecol..

[36] Baxter P. (2019). Markers of perinatal hypoxia-ischaemia and neurological injury: Assessing the impact of insult duration. Dev. Med. Child. Neurol..

[37] Salido-Vallejo R., Ruano J., Garnacho-Saucedo G., Godoy-Gijón E., Llorca D., Gómez-Fernández C., Moreno-Giménez J.C. (2014). Facial Angiofibroma Severity Index (FASI): Reliability assessment of a new tool developed to measure severity and responsiveness to therapy in tuberous sclerosis-associated facial angiofibroma. Clin. Exp. Dermatol..

[38] Safadi M.A., Berezin E.N., Farhat C.K., Carvalho E.S. (2003). Clinical presentation and follow up of children with congenital toxoplasmosis in Brazil. Braz. J. Infect. Dis..

[39] Giannattasio A., Bruzzese D., Di Costanzo P., Capone E., Romano A., D’Amico A., Bravaccio C., Grande C., Capasso L., Raimondi F. (2018). Neuroimaging Profiles and Neurodevelopmental Outcome in Infants With Congenital Cytomegalovirus Infection. Pediatr. Infect. Dis. J..

[40] Salel M., Tanchoux F., Viguier A., Cognard C., Larrue V., Bonneville F. (2013). Reversible bilateral basal ganglia lesions related to Epstein-Barr virus encephalitis. J. Neuroradiol..

[41] Al-Ansari A., Robertson N.P. (2019). Autoimmune encephalitis: Frequency and prognosis. J. Neurol..

[42] Pohl D., Alper G., Van Haren K., Kornberg A.J., Lucchinetti C.F., Tenembaum S., Belman A.L. (2016). Acute disseminated encephalomyelitis: Updates on an inflammatory CNS syndrome. Neurology.

[43] Wu X., Wu W., Pan W., Wu L., Liu K., Zhang H.L. (2015). Acute necrotizing encephalopathy: An underrecognized clinicoradiologic disorder. Mediators Inflamm..

[44] Ekici A., Yakut A., Yimenicioglu S., Bora Carman K., Saylısoy S. (2014). Clinical and Neuroimaging Findings of Sydenham’s Chorea. Iran. J. Pediatr..

[45] Aravamuthan B.R., Waugh J.L. (2016). Localisation of basal ganglia and thalamic damage in dyskinetic cerebral palsy. Pediatr Neur.

[46] Monbaliu E., Himmelmann K., Lin J.P., Ortibus E., Bonouvrié L., Feys H., Vermeulen R.J., Dan B. (2017). Clinical presentation and management of dyskinetic cerebral palsy. Lancet. Neurol..

[47] Préel M., Rackauskaite G., Larsen M.L., Laursen B., Lorentzen J., Born A.P., Langhoff-Roos J., Uldall P., Hoei-Hansen C.E. (2019). Children with dyskinetic cerebral palsy are severely affected as compared to bilateral spastic cerebral palsy. Acta Pediatr..

[48] Ozcan N., Ozcam G., Kosar P., Ozcan A., Basar H., Kaymak C. (2016). Correlation of computed tomography, magnetic resonance imaging and clinical outcome in acute carbon monoxide poisoning. Braz. J. Anesthesiol..

[49] Prockop L.D., Chichkova R.I. (2007). Carbon monoxide intoxication: An updated review. J. Neurosci. Sci..

[50] Marcucci G., Cianferotti L., Brandi M.L. (2018). Clinical presentation and management of hypoparathyroidism. Best. Pract. Res. Clin. Endocrinol. Metab..

[51] Albajara Sáenz A., Villemonteix T., Massat I. (2019). Structural and functional neuroimaging in attention-deficit/hyperactivity disorder. Dev. Med. Child. Neurol..

[52] Qiu A., Crocetti D., Adler M., Mahone M., Denckla Miller M.I., Mostofsky S.H. (2009). Basal Ganglia Volume and Shape in Children With Attention Deficit Hyperactivity Disorder. Am. J. Psychiatry.

[53] Subramanian K., Brandenburg C., Orsati F., Soghomonian J.J., Hussman J.P., Blatt G.J. (2017). Basal ganglia and autism—A translational perspective. Autism. Res..

[54] Mizuno Y., Kagitani-Shimono K., Jung M., Makita K., Takiguchi S., Fujisawa T.X., Tachibana M., Nakanishi M., Mohri I., Taniike M. (2019). Tomoda. Structural brain abnormalities in children and adolescents with comorbid autism spectrum disorder and attention-deficit/hyperactivity disorder. Transl. Psychiatry.

[55] Tonduti D., Panteghini C., Pichiecchio A., Decio A., Carecchio M., Reale C., Moroni I., Nardocci N., Campistol J., Garcia-Cazorla A. (2018). Encephalopathies with intracranial calcification in children: Clinical and genetic characterization. Orphanet. J. Rare Dis..

[56] Donzuso G., Mostile G., Nicoletti A., Zappia M. (2019). Basal ganglia calcifications (Fahr’s syndrome): Related conditions and clinical features. Neurol. Sci..

[57] Halefoglu A.M., Yousem D.M. (2018). Susceptibility weighted imaging: Clinical applications and future directions. World J. Radiol..

[58] Hodges K., Brewer S.S., Labbé C., Soto-Ortolaza A.I., Walton R.L., Strongosky A.J., Uitti R.J., van Gerpen J.A., Ertekin-Taner N., Kantarci K. (2016). RAB39B gene mutations are not a common cause of Parkinson’s disease or dementia with Lewy bodies. Neurobiol. Aging..

[59] Hussain S.A., Tsao J., Li M., Schwarz M.D., Zhou R., Wu J.Y., Salamon N., Sankar R. (2017). Risk of vigabatrin-associated brain abnormalities on MRI in the treatment of infantile spasms is dose-dependent. Epilepsia.

[60] Aschner J.L., Anderson A., Slaughter J.C., Aschner M., Steele S., Beller A., Mouvery A., Furlong H.M., Maitre N.L. (2015). Neuroimaging identifies increased manganese deposition in infants receiving parenteral nutrition. Am. J. Clin. Nutr..

[61] Shrot S., Poretti A., Tucker E.W., Soares B.P., Huisman T.A. (2017). Acute brain injury following illicit drug abuse in adolescent and young adult patients: Spectrum of neuroimaging findings. Neuroradiol. J..

